# Identification of Immunoglobulin G Autoantibody Against Malondialdehyde-Acetaldehyde Adducts as a Novel Serological Biomarker for Ulcerative Colitis

**DOI:** 10.14309/ctg.0000000000000469

**Published:** 2022-03-14

**Authors:** Michael J. Duryee, Rizwan Ahmad, Derrick D. Eichele, Carlos D. Hunter, Ananya Mitra, Geoffrey A. Talmon, Shailender Singh, Lynette M. Smith, Michael J. Rosen, Punita Dhawan, Geoffrey M. Thiele, Amar B. Singh

**Affiliations:** 1 Division of Rheumatology, Department of Internal Medicine, University of Nebraska Medical Center, Omaha, Nebraska, USA;; 2 Department of Biochemistry and Molecular Biology, University of Nebraska Medical Center, Omaha, Nebraska, USA;; 3 Division of Gastroenterology, Department of Medicine, University of Nebraska Medical Center, Omaha, Nebraska, USA;; 4 Department of Pathology and Microbiology, University of Nebraska Medical Center, Omaha, Nebraska, USA;; 5 Department of Biostatistics, College of Public Health, University of Nebraska Medical Center, Omaha, Nebraska, USA;; 6 Division of Pediatric Gastroenterology, Hepatology and Nutrition, Department of Pediatrics, Stanford University School of Medicine, Palo Alto, California, USA;; 7 Veterans Affairs Nebraska-Western Iowa Health Care System, Omaha, Nebraska, USA.

## Abstract

**INTRODUCTION:**

Inflammatory bowel disease (IBD) is associated with immune responses with oxidative stress wherein high levels of malondialdehyde result in the formation of a highly stable and immunogenic malondialdehyde-acetaldehyde adduct (MAA). Thus, this study evaluated the status of MAA and anti-MAA antibody isotypes in IBD and their potential as novel serological biomarkers for differentiating ulcerative colitis (UC) from Crohn's disease (CD).

**METHODS:**

Levels of MAA and anti-MAA antibodies were examined in patients with IBD (171), non-IBD gastrointestinal diseases (77), and controls (83) from 2 independent cohorts using immunohistochemistry and enzyme-linked immunosorbent assay. Receiver operating characteristic curves and Youden cutoff index from logistic regression were used to determine the sensitivity and specificity.

**RESULTS:**

The MAA and blood immunoglobulin G (IgG) anti-MAA antibody levels were significantly elevated in IBD compared with non-IBD patients (*P* = 0.0008) or controls (*P* = 0.02). Interestingly, patients with UC showed higher levels of IgG anti-MAA (*P* < 0.0001) than patients with CD including those with colonic CD (*P* = 0.0067). The odds ratio by logistic regression analysis predicted stronger association of IgG anti-MAA antibody with UC than CD. Subsequent analysis showed that IgG anti-MAA antibody levels could accurately identify (*P* = 0.0004) UC in the adult cohort with a sensitivity of 75.3% and a specificity of 71.4% and an area under the curve of 0.8072 (0.7121–0.9024). The pediatric cohort also showed an area under the curve of 0.8801 (0.7988–0.9614) and precisely distinguished (*P* < 0.0001) UC with sensitivity (95.8%) and specificity (72.3%).

**DISCUSSION:**

Circulating IgG anti-MAA antibody levels can serve as a novel, noninvasive, and highly sensitive test to identify patients with UC and possibly differentiate them from patients with CD.

## INTRODUCTION

Inflammatory bowel disease (IBD) is a group of chronic, progressive inflammatory disorders of the gastrointestinal tract constituted primarily of Crohn's disease (CD) and ulcerative colitis (UC). Both UC and CD are characterized by relapsing and remitting inflammation of the gut; however, despite the similarities, these diseases are diverse in their pathology and distribution. One key difference between the 2 diseases is that Crohn's affects the entire gastrointestinal tract, whereas UC affects only the colon. In the United States alone, approximately 3.1 million people suffer from IBD, and as many as 70,000 new cases of IBD are diagnosed each year, where approximately 20% of the patients have siblings who share a similar pattern of disease (1–3). The prevalence of IBD is higher in the developed western countries; however, newer epidemiologic studies suggest that the incidence of IBD is also on the rise in developing countries, including those in Asia, Africa, Eastern Europe, and South America (2,4,5). Causative factors remain unclear because the etiology of IBD is multifactorial and constitutes a complex interplay between intestinal microbiota, genetic susceptibility, the host's immune system, and environmental factors (5–10).

Importantly, chronically active inflammation is coupled directly to the generation of reactive oxygen species (ROS) from immune cells and serves as important physiological signaling molecules that contribute to immunological functions (11–13). However, excessive ROS and related products can be harmful, and continuous ROS release in the local mucosal microenvironment triggers collateral damage including extensive cellular and molecular damage, perpetuating intestinal inflammation, and mucosal injury (11–13). Most notably, an imbalance between the production and elimination of ROS characterizes oxidative stress, and accumulating evidence suggests that oxidative stress is at the crossroad of multiple factors that cause IBD (11–15). In recent years, several oxidative stress-relevant genetic risk loci, associated with IBD, have been identified and indisputably serve as the main trigger of neoplastic transformation in patients with IBD (13,16).

The lipid constituents of biological membranes are the primary targets of oxidative stress, and lipid peroxidation has been highlighted as a critical biological process driving the effects of oxidative stress involved in intestinal inflammation (17). Malondialdehyde (MDA), a lipid peroxidation product, is a naturally occurring immune adjuvant implicated in promoting autoimmunity and inflammation. Studies have now confirmed an elevated level of MDA in patients with IBD (18–22). Notably, MDA breaks down to form acetaldehyde and combines with MDA to form unique malondialdehyde-acetaldehyde adducts (MAAs), which can interact and modify biomolecules (23). Of note, MAA is highly stable and has been shown to promote inflammatory responses and cytokine secretion including tumor necrosis factor α, interleukin 6, and interferon γ (24). Recent studies have shown that MAAs may play a pathogenic role in the initiation/progression of chronic inflammatory pathologies including rheumatoid arthritis, alcoholic liver disease, and cardiovascular disease (24–27).

Animal studies have shown that MAA invokes both proinflammatory and profibrotic responses, suggesting that MAA may have a causal relationship with immunologic responses in the absence of an adjuvant (28,29). Previous studies have further shown that MAAs could generate antibody and T-cell responses to the carrier protein, providing a plausible mechanism by which tolerance to self-proteins is abolished, potentially resulting in autoimmunity (28,30). Accordingly, anti-MAA antibodies are upregulated in rheumatoid arthritis, alcoholic liver disease, and cardiovascular diseases (26,27,31). However, the status of the MAAs and anti-MAA antibodies in IBD remains unclear.

This study was undertaken to investigate the status of MAAs and the antibody responses to MAA in IBD and the specific correlation with UC and CD. Based on an extensive investigation using 2 independent cohorts of patients with IBD, we report here that the antibody responses to the MAAs could be used to discriminate patients with IBD from non-IBD patients, including patients with other autoimmune gastrointestinal diseases. We further demonstrate that immunoglobulin G (IgG) anti-MAA levels are highly specific to UC and may help differentiate UC from CD, including CD when restricted to the colon (colonic CD) with high specificity and sensitivity.

## MATERIALS AND METHODS

### Study design and patient recruitment

We performed a case-control study using 2 independent cohorts—1 adult and 1 pediatric/young adult—from 2 institutions. The first cohort was from the University of Nebraska Medical Center in Omaha and consisted of IBD and non-IBD adult patients and a control group with tissues and serum available through an institutional biorepository. Moreover, this cohort was part of a proof-of-principle study to determine whether anti-MAA antibody levels were increased in patients with IBD. Because the patients were from the Nebraska Biobank, all patient information except age, race, sex, and diagnosis were stripped from the samples and thus did not include any data on clinical manifestation of the patient's disease. The second cohort was a prospective cohort of pediatric and young adult IBD, non-IBD, and controls treated at Cincinnati Children's Hospital Medical Center, where well-annotated plasma samples were available. Non-IBD control patients in the pediatric cohort were individuals who underwent a clinically indicated lower endoscopy but did not have an IBD diagnosis and exhibited macroscopically and microscopically normal ileum and colon. Descriptive data were collected from the patients in both cohorts, with patient records deidentified. This study was approved by the institutional review board at both locations.

### Circulating blood anti-MAA immunoglobulin detection

An indirect (coated antigen) enzyme-linked immunosorbent assay was used to determine the levels of anti-MAA immunoglobulins in the blood (serum or plasma) from IBD, non-IBD, and controls described previously and briefly explained in the Supplementary Methods (see Supplementary Digital Content 1, http://links.lww.com/CTG/A770) (26).

### Immunofluorescence analysis

Immunofluorescence using antigen-specific antibody was used to detect MAA as described previously and explained in the Supplementary Methods (Supplementary Digital Content 1, http://links.lww.com/CTG/A770) (26).

### Statistical analysis

Patient characteristics and biomarkers were compared between diagnosis (UC, CD, irritable bowel syndrome [IBS], celiac disease, and controls) using the Kruskal-Wallis test and the Wilcoxon test for pairwise comparisons between the groups. Patients with IBS and celiac disease were grouped as non-IBD cohort. Adjustments for multiple comparisons were made using Bonferroni's method. Biomarker levels were highly skewed, so natural log transformations were taken before additional analysis and for display in the violin plots. Multivariate logistic regression was used to examine the markers and potential combinations as predictors of specific disease. Receiver operating characteristic (ROC) curves were used to determine optimal marker cut points, based on the Youden index, and estimates of area under the ROC curve (AUROC) and 95% confidence intervals (CIs) are given. To determine an optimal combination of biomarkers for identifying UC, CD, and control (3 groups simultaneously), recursive partitioning methods were used in a classification model (32). The decision trees were created using the party package: A Laboratory for Recursive Partitioning in the R version 3.2.0 programming language (33,34). *P* values <0.05 were considered statistically significant. Analysis was performed using SAS software, version 9.4 (SAS Institute, Cary, NC), R software, and Prism 9.0 (GraphPad Software, San Diego, CA) (35).

## RESULTS

### Cohort description

In this study, we used blood (serum/plasma) samples from 171 patients with IBD and 43 controls from 2 independent cohorts and IBD biopsy samples (n = 10/group). The adult cohort consisted of 102 patients with IBD (81 UC and 21 CD) and 25 controls (non-IBD patients). Descriptive characteristics of the cohort are summarized in Table 1. Differences between the patient ages in this cohort were adjusted in relevant analyses. The second pediatric cohort included younger individuals and was comprised of 69 patients with IBD (22 UC and 47 CD) and 18 control (non-IBD) patients (Table 1). An additional 50 IBS, 27 celiac disease patient samples (non-IBD patients), and 40 controls were further included in this study (see Supplementary Table 1, Supplementary Digital Content 6, http://links.lww.com/CTG/A775).

**Table 1.** Descriptive characteristics of the IBD patient's cohorts

	Cohort 1 (adult)			
	UC (n = 81)	CD (n = 21)	Control (n = 25)	*P* value
Age				
Median (IQR)	52.4 (37.6–64.4)	66.0 (56.0–72.0)	44.0 (36.1–59.4)	0.0058
Sex, n (%)				
Female Male	33 (41) 48 (59)	12 (57) 9 (43)	12 (48) 13 (52)	0.38
Race/ethnicity, n (%)				
Black Hispanic Other White	3 (4) 1 (1) 1 (1) 76 (94)	1 (5) 0 0 20 (95)	8 (32) 3 (12) 1 (4) 13 (52)	<0.001

	Cohort 2 (pediatric)			
	UC (n = 22)	CD (n = 47)	Control (n = 18)	*P* value
Age				
Median (range)	16.4 (7.7–21.3)	15.7 (5.6–21.7)	16.30 (9.0–18.0)	0.41
Sex, n (%)				
Female Male	14 (64) 8 (36)	16 (34) 31 (66)	10 (56) 8 (44)	0.047
Race/ethnicity, n (%)				
Black Hispanic White	1 (5) 1 (5) 20 (90)	3 (6) 1 (2) 44 (91)	3 (17) 0 15 (83)	0.55
Disease site, n (%)				
Colonic Ileal (L1) Ileocolonic (L3)	—	16 (34) 5 (11) 26 (55)	—	
Perianal involvement, fistula, n (%)				
No Yes	—	31 (65) 16 (34)	—	
Crohn's disease behavior, n (%)				
Nonstricturing and nonpenetrating Penetrating Stricturing	—	38 (83) 3 (7) 5 (11)	—	
Colitis classification, n (%)				
Extensive Left-sided Pancolitis Proctitis	3 (14) 3 (14) 14 (64) 2 (9)	—	—	
Oral 5-ASA, n (%)				
Yes	16 (73)	7 (15)	—	<0.001
Oral steroids, n (%)				
Yes	8 (36)	8 (17)	—	0.12
Rectal steroids, n (%)				
Yes	2 (9)	2 (4)	—	0.58
6-MP or azathioprine, n (%)				
Yes	3 (14)	13 (28)	—	0.23
Methotrexate, n (%)				
Yes	1 (5)	2 (4)	—	1.0
Antibiotic, n (%)				
Yes	0	6 (13)	—	0.17
Anti-TNF biologic, n (%)				
Yes	5 (22)	13 (28)	—	0.77
Antileukocyte trafficking biologic, n (%)				
Yes	0	2 (4)	—	1.0

5-ASA, 5-aminosalicylic acid; 6-MP, 6-mercaptopurine; CD, Crohn's disease; IBD, inflammatory bowel disease; IQR, interquartile range; TNF, tumor necrosis factor; UC, ulcerative colitis.

### Blood IgG anti-MAA levels are increased in patients with IBD

**Figure 1.**
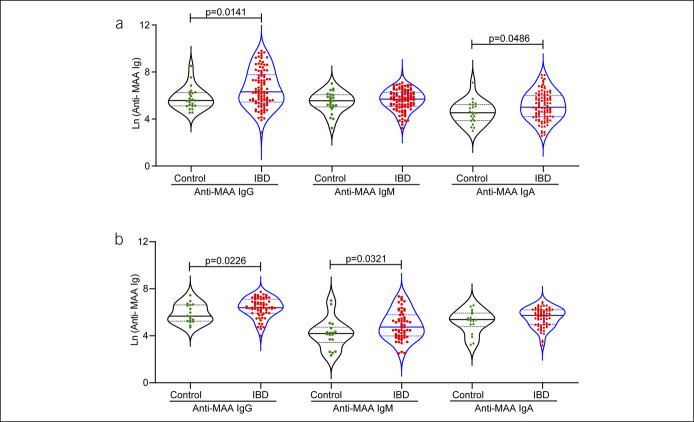
Differential levels of anti-MAA immunoglobulin isotypes were detected in patients with IBD compared with control individuals: ELISA immunoassay was used to measure the blood level of anti-MAA immunoglobulin isotypes. Data were transformed into a natural log scale, and violin plots were used to demonstrate the differences among the groups. (**a**) Serum level of anti-MAA antibodies in the adult cohort and (**b**) plasma level of anti-MAA immunoglobulins in a pediatric cohort. ELISA, enzyme-linked immunoassay; IBD, inflammatory bowel disease; MAA, malondialdehyde-acetaldehyde adduct.

Oxidative stress promotes susceptibility to IBD and disease severity. MDA, a lipid peroxidation product, readily combines with acetaldehyde to form MAA, which is highly stable and has been shown to be increased in certain autoimmune inflammatory diseases (17). Therefore, we examined the status of blood anti-MAA immunoglobulins in the adult cohort of patients with IBD. As shown in Figure 1a, the IgG and IgA anti-MAA antibody levels were significantly upregulated in patients with IBD (vs controls; *P* = 0.0141; *P* = 0.0486). IgM anti-MAA antibody levels were not significantly different. We then used an independent IBD pediatric patient cohort from a different institution to validate these findings.

Similar to the adult cohort, we found significantly higher IgG anti-MAA antibody levels when compared with controls in the pediatric cohort (*P* = 0.0226; Figure 1b). In addition, the IgM but not IgA anti-MAA antibody levels were significantly upregulated in this cohort (*P* = 0.0321). Overall, data from both cohorts revealed a consistent upregulation in blood IgG anti-MAA immunoglobulins in patients with IBD compared with controls.

### IgG anti-MAA levels differentiate patients with IBD from non-IBD patients

Based on the above findings, we further examined whether IgG anti-MAA antibody levels can also differentiate patients with IBD from patients with other inflammatory and noninflammatory gastrointestinal disorders. We examined IgG anti-MAA antibody levels in patients with IBS and celiac diseases. As shown in Supplementary Figure 1 (see Supplementary Digital Content 2, http://links.lww.com/CTG/A771), anti-MAA autoantibody levels were significantly increased in IBD compared with non-IBD gastrointestinal diseases (*P* = 0.0074). Overall, the data revealed that the high IgG anti-MAA level distinguishes IBD from those without gastrointestinal diseases and patients with other inflammatory and noninflammatory gastrointestinal diseases.

### Increased blood levels of IgG anti-MAA antibodies in patients with IBD are highly specific to UC

**Figure 2.**
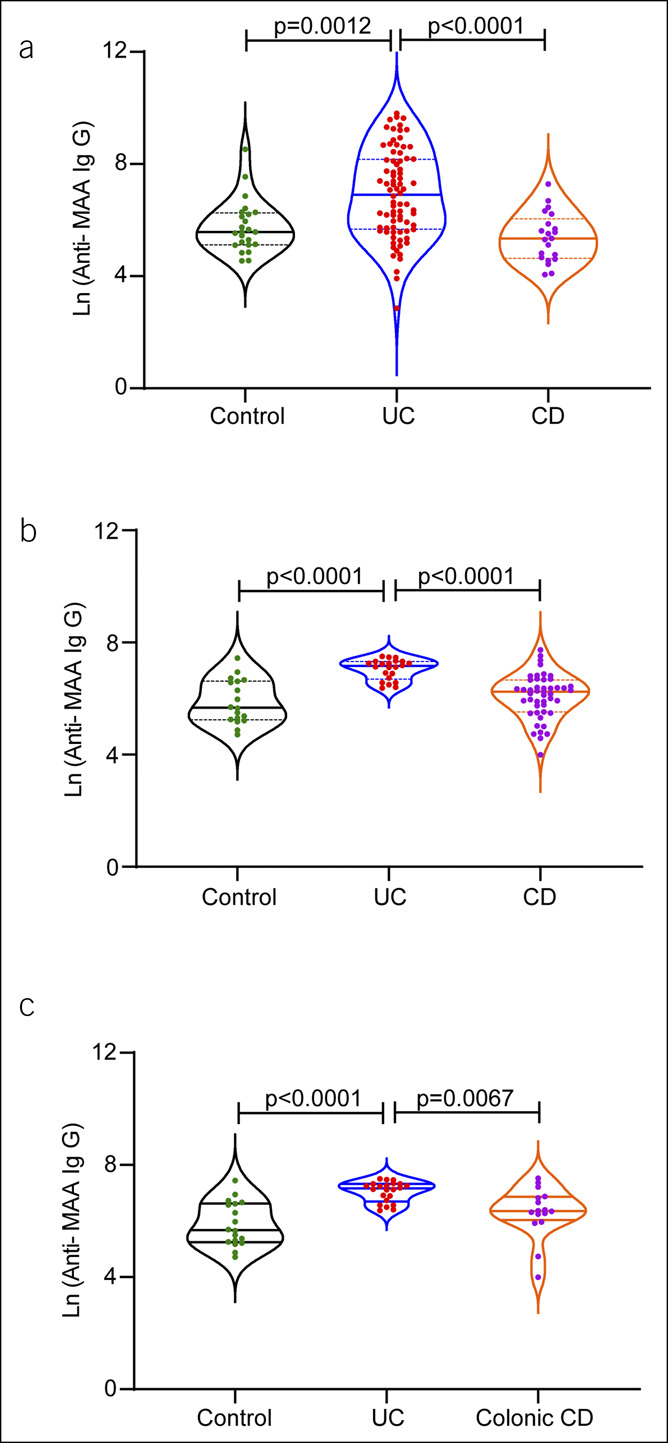
IgG Anti-MAA antibody precisely differentiates patients with UC from patients with CD: ELISA quantified blood IgG antibodies against MAA and data were presented as a natural log-transformed scale. (**a** and **b**) Comparative serum/plasma IgG anti-MAA antibody analysis in both adult and pediatric cohorts. (**c**) Blood anti-MAA IgG level significantly stratified UC from colonic CD. CD, Crohn's disease; ELISA, enzyme-linked immunoassay; IgG, immunoglobulin G; MAA, malondialdehyde-acetaldehyde adduct; UC, ulcerative colitis.

UC and CD are the principal but diverse subtypes of IBDs (36). Based on differing pathobiology of UC and CD, we further investigated whether the observed increase in blood IgG anti-MAA levels is specific for 1 subtype or is similar in both diseases. To assess the specificity of anti-MAA antibodies in classifying patients with IBD into subtypes, we grouped patients with IBD into UC and CD subtypes. Next, we compared IgG anti-MAA levels in patients with UC, CD, and controls in a pairwise analysis, with adjusting for multiple comparisons (Figure 2a). This analysis suggested that the blood IgG anti-MAA levels were significantly increased in patients with UC than in patients with CD and controls (Figure 2a; *P* < 0.0001; *P* = 0.0012). No significant differences were found in IgM and IgA levels in UC compared with CD (see Supplementary Figure 2A and B, Supplementary Digital Content 3, http://links.lww.com/CTG/A772). Similar to the adult cohort, only IgG anti-MAA antibody levels were significantly increased when comparing patients with UC with control and patients with CD in the pediatric cohort (Figure 2b; *P* < 0.0001). The IgM anti-MAA was found to be significantly different only in UC vs controls (*P* = 0.0141; *P* = 0.0451) (see Supplementary Figure 2C and D, Supplementary Digital Content 3, http://links.lww.com/CTG/A772).

### IgG anti-MAA antibody levels are specific to UC colon inflammation

The above data (Figure 2a and b) showed increased serum/plasma levels of IgG anti-MAA antibodies in UC, which is localized to the colon. By contrast, the CD can affect any portion of the intestinal tract from the mouth to the anus. However, in approximately 15%–20% of patients with CD, the disease is localized only to the colon, which can be a critical confounding factor in the accurate diagnosis of CD or UC (37). Twenty-five percent of children with CD exhibit colon-only involvement, with no small intestinal inflammation; colon-only phenotype occurs even more frequently in children aged less than 10 years, accounting for 40% of CD in this younger age group (38–40). To examine whether observed increases in IgG anti-MAA antibody are specific to UC or over any colonic inflammation, we further analyzed the blood levels of IgG anti-MAA antibodies among controls, UC, and CD patients with colon-only involvement from the pediatric cohort. Interestingly, the IgG anti-MAA levels were significantly higher in UC even compared with CD with colon-only involvement (Figure 2c; *P* = 0.0067). Overall, these results indicated that increased serum IgG anti-MAA levels in IBD are specific to UC colon inflammation.

### The MAAs are robustly upregulated in UC

**Figure 3.**
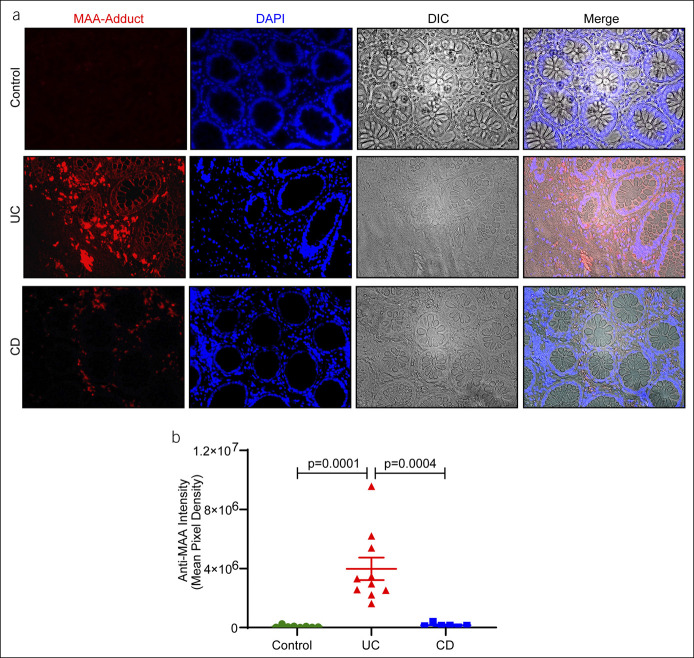
MAA is significantly upregulated in patients with UC: Immunofluorescence analysis of MAA was performed in the biopsy samples from patients with UC and Crohn's disease and normal colon (10 biopsies/phenotype). (**a** and **b**) Representative images and quantitative analysis of the signal intensity of MAA. Values are presented as mean + SEM. MAA, malondialdehyde-acetaldehyde adduct; UC, ulcerative colitis.

Having uncovered a novel finding of a specific increase in anti-MAA IgG in patients with UC, we considered the MAA for the immunogenic potential that triggers anti-MAA IgG production. Therefore, we examined whether biopsy samples from patients with UC had high expression of MAA. De-identified specimens from normal colon and biopsies from patients with UC and CD were obtained from the UNMC pathology archives. As shown in Figure 3a, a substantial MAA was found in the IBD biopsy samples compared with controls. However, UC patients' biopsy sections reacted more robustly to the anti-MAA antibody compared with the biopsies from the patients with CD. Staining intensity analysis confirmed that the mean pixel density of the antibody reactivity increases significantly in UC compared with CD (Figure 3b; *P* = 0.0004). These data reflect excessive lipid peroxidation in the patients with UC.

### Blood IgG anti-MAA antibody levels identify UC over CD

We found increased levels of both MAAs and anti-MAA antibodies in patients with UC vs patients with CD. Therefore, we further performed binary logistic regression analysis to examine the association potential of immunoglobulin isotypes in the diagnosis of UC from CD. The results from the adult cohort revealed a significant association with UC of the IgG antibody isotype alone (odds ratio [OR] 2.38; 95% CI: 1.47–3.83, *P* = 0.0004) or with the addition of IgA and IgM antibody isotype (OR 2.69; 95% CI: 1.51–4.79, *P* = 0.0007 and OR 2.83; 95% CI: 1.64–4.89, *P* = 0.0002; Table 2). Interestingly, the outcomes from the pediatric cohort showed an even stronger association of IgG anti-MAA antibodies with UC over CD with increased OR (OR 17.24; 95% CI: 4.2–70.78, *P* < 0.0001) and with the addition of IgA and IgM antibodies (OR 18.33; 95% CI: 3.91–85.99, *P* = 0.0002 and OR 27.57; 95% CI: 4.65–163.69, *P* = 0.0003; Table 3). Overall, the blood anti-MAA IgG level showed firm association with UC.

**Table 2.** Adult cohort logistic regression analysis

	OR	Lower CI	Upper CI	*P* value	AUC	Lower CI	Upper CI	Cutpoint^a^	Sensitivity	Specificity
Ln IgG	2.375	1.474	3.828	0.0004	0.8072	0.7121	0.9024	Ln(IgG) >5.711 IgG >166.9	0.753	0.714
Ln IgM	0.969	0.555	1.691	0.91	0.4951	0.3406	0.6497	Ln(IgM) <6.86 IgM <953.6	0.974	0.190
Ln IgA	1.333	0.870	2.043	0.19	0.6019	0.4568	0.7470	Ln(IgA) >5.37 IgA >214.5	0.506	0.800
Ln IgG Ln IgA	2.694	1.514	4.792	0.0007	0.7949	0.6949	0.8949	Pr(UC) >0.855	0.557	0.950
	0.748	0.432	1.296	0.30						
Ln IgG Ln IgM	2.827	1.635	4.888	0.0002	0.8156	0.7246	0.9067	Pr(UC) >0.823	0.641	0.905
	0.641	0.350	1.175	0.15						

Logistic regression analysis revealed that serum IgG anti-MAA levels significantly separate the patients with UC from CD in the adult cohort.

AUC, area under the curve; CD, Crohn's disease; CI, confidence interval; IgG, immunoglobulin G; IgM, immunoglobulin M; MAA, malondialdehyde-acetaldehyde adduct; OR, odds ratio; UC, ulcerative colitis.

a Predict UC if condition is met.

**Table 3.** Pediatric cohort logistic regression analysis

	OR	Lower CI	Upper CI	*P* value	AUC	Lower CI	Upper CI	Cutpoint^a^	Sensitivity	Specificity
Ln IgG	17.242	4.200	70.779	<0.0001	0.8801	0.7988	0.9614	Ln(IgG) >6.406 IgG >605.8	0.955	0.723
Ln IgM	1.563	0.983	2.485	0.059	0.6772	0.5424	0.8119	Ln(IgM) >4.333 IgM <76.1	0.909	0.487
Ln IgA	2.281	1.040	5.001	0.040	0.6466	0.5121	0.7810	Ln(IgA) >5.171 IgA >176.1	0.905	0.400
Ln IgG Ln IgA	18.332	3.908	85.995	0.0002	0.8921	0.8154	0.9688	Pr(UC) >0.260	0.952	0.756
	2.996	0.953	9.421	0.061						
Ln IgG Ln IgM	27.573	4.645	163.69	0.0003	0.9021	0.8177	0.9865	Pr(UC) >0.471	0.909	0.872
	2.252	1.080	4.698	0.030						

Logistic regression analysis of plasma IgG anti-MAA levels discriminate patients with UC from CD in a pediatric cohort.

AUC, area under the curve; CD, Crohn's disease; CI, confidence interval; IgG, immunoglobulin G; IgM, immunoglobulin M; MAA, malondialdehyde-acetaldehyde adduct; OR, odds ratio; UC, ulcerative colitis.

a Predict UC if condition is met.

### Discriminating power of blood IgG anti-MAA antibodies in differentiating UC from CD

**Figure 4.**
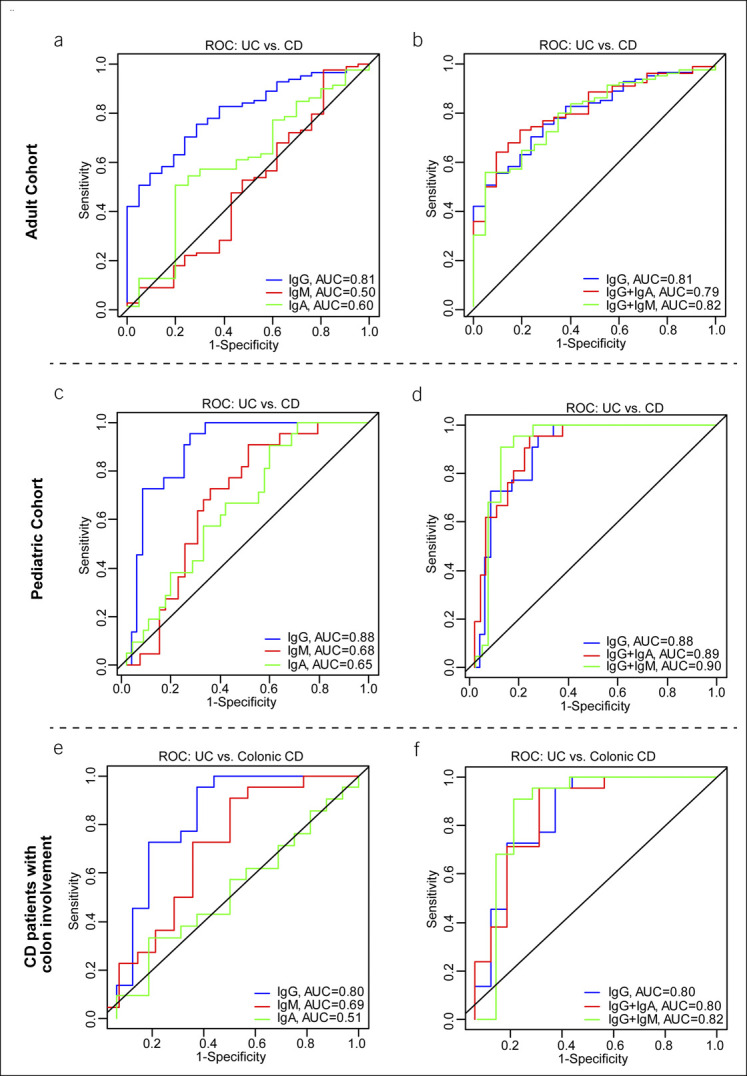
AUROC curves support the diagnostic performance of IgG anti-MAA antibody as a biomarker for identifying UC and differentiating from CD: ROC curve analysis by logistic regression indicates the predictive power of circulating IgG anti-MAA compared with IgA and IgM. (**a** and **b**) ROC analysis of IgG anti-MAA in association with IgA and IgM indicates the discriminating potential of IgG anti-MAA antibody in separating UC from CD in the adult cohort. (**c** and **d**) ROC curve analysis showed significant discrimination of UC from CD in the pediatric cohort. (**e** and **f**) ROC curve by logistic regression analysis significantly predicts UC on CD with colon-only involvement. AUROC, area under the receiver operating characteristic; CD, Crohn's disease; IBD, inflammatory bowel disease; IgA, immunoglobulin A; IgG, immunoglobulin G; MAA, malondialdehyde-acetaldehyde adduct; ROC, receiver operating characteristic; UC, ulcerative colitis.

We further examined the discriminating potential of immunoglobulin isotypes in the diagnosis of UC from CD using the ROC curve approach. As shown in Figure 4a and Supplementary Figure 3A and B (see Supplementary Digital Content 4, http://links.lww.com/CTG/A773), blood IgG anti-MAA antibody levels have significant power to separate UC from CD and controls. Notably, the IgG anti-MAA antibody had a significantly higher AUROC (AUROC 0.8072; 95% CI: 0.7121–0.9024) with a sensitivity of 75.3% and a specificity of 71.4% than any other Ig isotypes tested (Table 2). The results from IgM and IgA isotypes by using logistic regression analysis revealed that the levels of these isotypes could not distinguish UC from CD (*P* = 0.19; *P* = 0.91; Table 2). The addition of the IgM values further increased the AUROC value of IgG anti-MAA, however, not significantly over IgG anti-MAA alone (Table 2; Figure 4b; see Supplementary Figure 3C and D, Supplementary Digital Content 4, http://links.lww.com/CTG/A773). However, the addition of IgA to IgG did not increase AUROC compared with IgG alone. Overall, these results suggested that blood IgG anti-MAA antibodies can discriminate UC from CD with high sensitivity and specificity.

Similar results were obtained when ROC analysis was performed on the data from the pediatric cohort (UC vs controls and CD vs control; Figure 4c; see Supplementary Figure 3C and D, Supplementary Digital Content 4, http://links.lww.com/CTG/A773). ROC curve analysis showed that IgG anti-MAA antibody was the strongest predictor of UC diagnosis over CD with an AUROC of 0.8801 (95% CI: 0.7988–0.9614, *P* < 0.0001), a sensitivity of 95.5%, and a specificity of 72.3% (Table 3; Figure 4c; see Supplementary Figure 4A and B, Supplementary Digital Content 5, http://links.lww.com/CTG/A774). The IgA anti-MAA antibody was also a significant predictor of UC (*P* = 0.04) albeit with a poor specificity of 40.0% (Table 3). The IgM anti-MAA values were trending toward significance (*P* = 0.059) for the diagnosis of UC; however, specificity was poor (48.7%), making it an unlikely candidate as a useful biomarker (Table 3). The discriminating potential for IgG anti-MAA AUROC was increased with the addition of IgG and IgM. The AUROC for IgG + IgM and +IgA was 0.8921 and 0.9021, respectively; however, this increase was not statistically significant compared with IgG alone (Table 3; Figure 4d; see Supplementary Figure 4C and D, Supplementary Digital Content 5, http://links.lww.com/CTG/A774). Because CD with colon involvement can be difficult to separate from UC, we also analyzed samples from pediatric patients with colonic CD compared with UC. As shown in Figure 4e and f, AUROC of IgG anti-MAA antibody was also discriminatory for UC from the CD with colon-only involvement.

**Figure 5.**
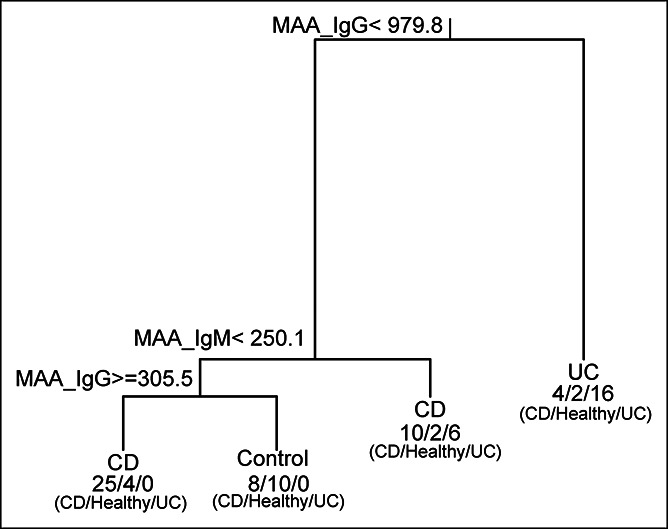
Decision tree supports the interpretation of IgG anti-MAA biomarker in IBD: decision tree showing the accuracy of IgG anti-MAA to predict UC from CD. CD, Crohn's disease; IBD, inflammatory bowel disease; IgG, immunoglobulin G; MAA, malondialdehyde-acetaldehyde adduct; UC, ulcerative colitis.

Furthermore, to discriminate the 3 groups simultaneously (UC, CD, and control) a classification tree approach was used forming a decision tree in the pediatric cohort based on the anti-MAA markers (Figure 1b). The outcome suggested that at blood IgG anti-MAA antibody level ≥979.8, UC is predicted. By contrast, CD is anticipated if the IgG anti-MAA level is <979.8 with ≥250.1 IgM anti-MAA. If IgM anti-MAA is <250.1 and IgG anti-MAA is between 305.5 and 979.8, then CD is the most likely diagnosis. Finally, if IgM anti-MAA is <250.1 and IgG anti-MAA is <305.5, then there is no disease present, and one would consider controls as the diagnosis (Figure 5). The decision tree showed an overall accuracy of 69% in predicting the 3 groups.

Overall, the results from these 2 independent cohorts and IBD biopsies suggest that IgG anti-MAA displayed a better discriminatory performance over IgM and IgA anti-MAA antibodies in identifying UC and differentiating from CD. Our data may help to delineate a clinical utility of MAAs and IgG anti-MAA antibodies in the diagnosis of UC, especially when it comes to distinguishing UC from CD localized to the colon.

## DISCUSSION

In this study, we provide conclusive evidence that MAA and anti-MAA immunoglobulin responses are significantly upregulated in patients with IBD than non-IBD gastrointestinal diseases. Our comprehensive analysis further demonstrates that the IgG anti-MAA levels specifically can identify patients with UC with high sensitivity and specificity and differentiate them from the patients with CD even when CD is confined to the colon. In this regard, a meta-analysis found pANCA to discriminate UC from CD with a sensitivity, specificity, and area under the curve of 55.3%, 88.5%, and 0.81, respectively, with significant between-study heterogeneity (41). Furthermore, commercially available serological biomarkers including anti-saccharomyces cerevisiae antibody (ASCA) IgA, ASCA IgG, anti-outer membrane protein C (OmpC), anti-flagellin (CBir1), anti-neutrophil cytoplasmic antibody (ANCA), and peripheral antineutrophil cytoplasmic antibodies (pANCA) found the area under the curve for CD vs UC to be 0.78 (42). In comparison, our pediatric cohort demonstrated IgG anti-MAA antibodies having a sensitivity of 95.5%, a specificity of 72.3%, and an AUROC of 0.8801 (95% CI: 0.7988–0.9614, *P* < 0.0001) in differentiating UC from CD.

Importantly, increased oxidative stress and MDA have been reported in several chronic inflammatory diseases, including IBD; however, the outcomes are variable primarily due to the fact that MDA is not very stable (43,44). However, to the best of our knowledge, this is the first report suggesting MDA-derived and highly stable MAA formation is significantly increased in patients with IBD. These results are in line with those obtained by others who measured elevated MAAs and suggested their importance in chronic diseases, including rheumatoid arthritis, alcoholic liver disease, lung injury, and cardiovascular disease (24–27). Of note, a significant immune reactivity of the anti-MAA antibody was also observed in the biopsy samples primarily from the patients with UC. Although studies have shown that MDA concentrations can be elevated in both, the patients with UC and CD compared with normal (45), it may be a possibility that the production of MAAs in UC and CD is not solely dependent on the oxidative stress and rather dependent on the differential antioxidant responses in these 2 subtypes of IBDs (46). Another possibility for the higher IgG anti-MAA antibody formation in UC could be differential microbiota in UC leading to differential T-cell responses and extensive epithelial damage. In this regard, we have recently described differential gut microbiota colonization in patients with UC vs patients with CD (47). In addition, as previously reported, disproportionate cytokine levels and T-helper 2 responses would suggest B-cell activation that could cause an increase in IgG antibodies in UC (48–50). Of note, IgG1 autoantibodies reactive to colonic epithelial cells are often detected in the sera of patients with UC than CD (51,52). Thus, it seems that the inflammatory evolves through diverging pathways in CD and UC. Recent studies using single-cell analysis of UC and CD biopsies have further highlighted the inherent heterogeneity of UC and CD and the limitations of the current diagnostic assays (53). Importantly, this study unveils that MAA formation could play an important role in IBD pathogenesis, more specifically in UC. However, the causal undertakings remain to be examined and part of our ongoing studies.

Notably, MAA is recognized as a terminal and stable MDA adduct that is highly immunogenic and initiates strong innate and acquired responses (23,28). Studies have further suggested that an increase in anti-MAA antibodies has a major influence on certain inflammatory disease states (23–26,30). However, the reactivity of one isotype of immunoglobulins over another to the MAA would indicate a highly unique immune response (23,26,28). In this study, we begin to fill that gap by reporting that blood IgG anti-MAA antibody is preferentially developed over IgM and IgA in patients with IBD. Remarkably, both adult and pediatric cohorts showed a significant increase in IgG anti-MAA isotype over IgM and IgA in patients with IBD than healthy controls despite the age differences in the patient cohorts. Our findings are consistent with earlier reports that suggest a rise in IgG serum levels in other inflammatory diseases (23–26,28). Notably, a recent study by Smillie et al. (54) has identified patients with IBD expressing unique cellular modules in their inflamed tissues consisting of the IgG plasma cells. Subsequently, circulating levels of anti-MAA immunoglobulin have been shown to correlate with the extent of tissue damage in acute injury and chronic disease states (55). Specific switching of the immune response, an IgG anti-MAA antibody response over other isotypes in IBD could be due to the extent and duration of chronic injury, inflammation, and cytokine milieu compared with normal (56). In addition, the literature has shown that IgM is initially produced on contact with new or “acute” antigens and then switches to IgG on chronic or repeated exposure to that same antigen (57). Thus, the reactivity of the IgG isotype to MAAs would indicate a chronic and highly specific immune response. It would be interesting to compare patients with acute, active inflammation in a UC flare as compared with patients in histologic remission to determine whether there is a different signal IgG vs IgM, and this is part of our ongoing studies.

Interestingly, the individuals from these 2 cohorts belong to 2 different age groups (adult and pediatric/young adult). However, a similar increase in the magnitude of blood IgG levels in both old and younger individuals was observed, indicating that anti-MAA IgG is generated during UC development, regardless of age, and may serve as a useful biomarker for all patients with UC (58,59). Notably, our analysis excludes the possibility that the observed increase in IgG anti-MAA antibody is simply a product of colonic inflammation because our IgG anti-MAA antibody levels also differentiated patients with UC from patients with CD where the disease was localized only to the colon. Our findings of increased MAA specifically in the colon tissues of patients with UC corroborate that the role of MAAs in chronic intestinal inflammation is likely specific to UC over CD.

However, despite the novelty of our findings, we concede that this study has certain limitations. Currently, we do not know the mechanism/s for the increased levels of anti-MAA IgG in patients with UC; however, such studies are part of our ongoing studies. The lack of the known association of anti-MAA IgG with the disease activity indices is yet another limitation and remains part of our future studies. At this time, we can also not rule out the confounding effects of therapeutic modalities, disease progression, and factors, such as obesity, smoking, and alcohol consumption on current findings. Irrespectively, we consider the results in these studies as significant and promising. Of note, seroreactivity to microbial antigens in UC and CD (e.g., pANCA, ASCA, and CBIR) does not correlate with disease activity. Nonetheless, these antibodies have proven useful for diagnosis and prognosis and are still being investigated regarding their role in disease pathogenesis. We are currently engaged in a prospective study in patients with IBD to determine the association of the anti-MAA IgG with patients with UC and CD in association with the disease activity indices and therapeutics.

In summary, our study indicates that increased levels of the MAA and the development of IgG antibodies to this adduct are direct and useful markers for oxidative stress-mediated tissue injury and immune response in patients with IBD. This study suggests that there is an increased immune reactivity to MAA in UC compared with CD. In addition, this study indicates that IgG anti-MAA antibodies have the potential for the development as a clinical peripheral blood biomarker for distinguishing UC from CD with improved diagnostic accuracy compared with currently approved and accepted serological biomarkers (41,42). Our results justify future comprehensive studies to understand the underlying mechanisms and diagnostic significance of MAAs and immune reactivity in UC.

## CONFLICTS OF INTEREST

**Guarantor of the article:** Amar B. Singh, PhD.

**Specific author contributions:** The first 2 authors contributed equally to this work, while the last 2 authors are cocorresponding authors. M.J.D., R.A., A.M., C.D.H., and M.J.R.: conducted data acquisition. A.B.S. and G.M.T.: conceptualized the study. G.A.T.: performed pathological evaluations. L.M.S.: performed statistical analyses. A.B.S., R.A., M.J.D., and G.M.T.: wrote the manuscript. R.A., L.M.S., M.J.R., D.D.E., S.S., P.D., A.B.S., and G.M.T.: critically reviewed manuscript. All authors approved the final draft before the manuscript was submitted.

**Financial support:** This work was supported by grants from the Veterans Affairs (Merit Grant) BX002761B (A.B.S.) and BX002086 (P.D.); the National Institutes of Health under Award Numbers DK124095 (A.B.S.), CA216746 (P.D.), and DK117119 (M.J.R.); Department of Internal Medicine Funds (G.M.T.); and U54GM115458/NIGMS NIH HHS/United States, the Great Plains IDeA-CTR grant, and the Nebraska Research Initiative.

**Potential competing interests:** None to report.

**Study Highlights**

**WHAT IS KNOWN**

✓ Role of oxidative stress in promoting IBD is widely recognized.
✓ Malondialdehyde (MDA), a lipid peroxidation product, reacts with acetaldehyde and forms a unique Malondialdehyde-Acetaldehyde Adduct (MAA).
✓ However, the role of MAA-modification and/or anti-MAA antibodies in IBD has not been examined.

**WHAT IS NEW HERE**

✓ The level of MAA-adducts and anti-MAA IgG are significantly increased in IBD compared control.
✓ Anti-MAA Ig G can accurately discriminate Ulcerative Colitis patient from Crohn's Disease with high specificity and sensitivity.
✓ Circulating IgG anti-MAA auto-antibody levels can serve as a novel, non-invasive and highly sensitive biomarker for differentiating Ulcerative Colitis patient from Crohn's Disease.

## Supplementary Material

SUPPLEMENTARY MATERIAL
