# Supplementary material for: Identification of Immunoglobulin G Autoantibody Against Malondialdehyde-Acetaldehyde Adducts as a Novel Serological Biomarker for Ulcerative Colitis
Source: Clin Transl Gastroenterol. 2022 Mar 14;13(4):e00469. doi: 10.14309/ctg.0000000000000469 (PMC9038499; doi:10.14309/ctg.0000000000000469)
Supplement: SUPPLEMENTARY MATERIAL [file ct9-13-e00469-s001.docx]

**Supplemental Methods:**

Human Subjects: Blood was collected in EDTA tubes, processed by density gradient separation at UCSD or LJI, and cryopreserved in 10% DMSO in heat inactivated fetal bovine serum and stored in liquid nitrogen until ready to use.

ELISAs: Recombinant proteins for Spike and the RBD were provided by the Saphire laboratory as previously described (1–3). Recombinant nucleocapsid protein was purchased from Genscript. Corning 96-well half area plates were coated with 1$\mu$g/mL of recombinant protein in phosphate buffered saline (PBS) overnight at 4$^{\circ}$C. The next day, plates were blocked with PBS containing 3% milk ad 0.05% Tween-20 for 1.5 hours at room temperature (RT). All plasma was heat inactivated at 56$^{\circ}$C for 30-60 minutes prior to addition to the plates. Plasma was diluted in PBS containing 1% milk and 0.05% Tween-20 and incubated at RT for 1.5 hours. Plates were washed 5x with 0.05% PBS-Tween. Anti-human IgG peroxidase antibody produced in goat (Sigma A6029) was used at 1:5000 dilution in PBS containing 1% milk and 0.05% Tween-20. Plates were washed 5x with 0.05% PBS-Tween and developed using TMB Substrate kit (ThermoScientific) at room temperature. The reaction was stopped with 2M sulfuric acid. Plates were read on a Spectramax Plate Reader at 450nm using SoftMax Pro. A positive control standard was used by pooling plasma from 6 convalescent subjects to normalize between experiments. Endpoint titers were plotted for each sample using background subtracted data and calculated at the dilution which gives a reading above the limit of detection of 0.1. The limit of detection was set at 3 based on the lowest dilution of plasma used.

Neutralizing Antibody Titers: Vero cells were seeded in 96-well plate to produce a monolayer at time of infection, as previously described (2,3). Pre-titrated rVSV-SARS-CoV-2 (Spike pseudotyped VSV-$\Delta$G-GFP) were incubated with serially diluted human plasma at 37$^{\circ}$C for 60 minutes prior to addition to confluent Vero cell monolayers. Cells were incubated for 12-16 hours at 37$^{\circ}$C I 5% CO_2_, fixed with 4% paraformaldehyde, stained with 1$\mu$g/mL of Hoescht, and imaged using a Cellnsight CX5 imager to quantify total number of cells expressing GFP. The limit of detection was set at 19 based on lowest dilution of plasma. Neutralization IC_50_ titers were calculated using One-Site Fit Log IC_50_ regression in GraphPad Prism 8.0.

Antigen-specific memory B cells: As previously described (3), biotinylation of full-length Spike and RBD recombinant proteins was performed using biotin-protein ligase standard reaction kit (Avidity, Cat # Bir500A) and dialyzed overnight against PBS. Biotinylated Spike was mixed with Streptavidin BV421 (Biolegend 405225) and Streptavidin Alexa Fluor 647 (ThermoFisher S21374) at 20:1 ratio (~ 6:1 molar ratio). Biotinylated RBD was mixed with streptavidin PE/Cyanine 7 (Biolegend 405225) at 2.2:1 ratio (~ 4:1 molar ratio). The antigen probes were mixed in Brilliant Buffer (BD Bioscience 566349) containing 5 $\mu$M free d-Biotin to ensure minimal cross-reactivity of antigen probes. PBMCs were stained with the antigen probe cocktail at 4$^{\circ}$C for 60 minutes prior to surface staining. Dead cells were stained using LIVE/DEAD Fixable Blue Stain kit (ThermoScientific L34962) at 4$^{\circ}$C for 30 minutes.

Antibodies included: CD62L (BUV395, Clone SK11), CD19 (BUV563, Clone SJ25C1), CD307 (BU615, Clone 509F6), CD95 (BUV737, Clone DX2), CXCR3 (BUV805, Clone 1 C6/CXCR3), IgD (BV510, Clone IA6-2), IgM (BV570, Clone MHM-88), CD24 (BV605, Clone ML5), CD20 (BV650, Clone 2H7), CXCR5 (BV750, Clone RF8B2), CD71 (BV786, Clone M-A712), CD27 (BB515, Clone M-T271), IgA (VioBright FITC, Clone IS11-8E10), CD3 (PerCP, Clone Sk7), CD14 (PerCp, Clone 63D3), CD16 (PerCp, Clone 3G8), CD56 (PerCp, Clone HCD56), IgG (PerCPCy5.5, Clone M1310G05), CD85 (PE/Dazzle 594, Clone GHI/75), CD11c (PECy-5, Clone 3.9), CD21 (AF700, Clone Bu32), CD38 (APC/Fire 810, Clone HIT2), Streptavidin BV421, Streptavidin 721, Streptavidin PE, Streptavidin PECy5.5, Streptavidin Alexa Fluor 647).

Cells were acquired on Cytek Aurora CS and analyzed using FlowJo 10.7.1.

Immunophenotyping: PBMCs were stained for B cells and T cell subsets.

Antibodies for B cells included: Live/Dead e780, CD3 (APC-eFluor 780, Clone OKT3), CD14 (APC-eFluor 780, Clone 61D3), CD16 (APC-eFluor 780, Clone CB16), CD19 (AF700, Clone H1B19), IgD APC, IgM (BV421, Clone G20-127), CD20 (BV570, Clone 2H7), IgG (BV650, Clone G18-145), IgA (FITC, Clone IS11-8E10), CD27 (PerCPe710, Clone O323), CD38 (Pe-Cyanine7, Clone HIT2).

Antibodies for T cells included: Live/Dead e780, CD19 (APC-eFluor 780, Clone HIB19), CD14 (APC-eFluor 780, Clone 61D3), CD16 (APC-eFluor 780, Clone CB16), CD8 (AF700, Clone RPA-T8), CD4 (APC, Clone OKT4), CXCR5 (BV421, Clone J25D4), CD45RA (BV570, Clone HI100), CXCR3 (BV650, Clone G025H7), PD-1 (BV785, Clone EH12.2H7), CCR7 (FITC, Clone G043H7), CCR4 (PE, Clone 161), ICOS (PerCPe710, Clone ISA-3), CCR6 (Pe-Cyanine7, Clone 11A9).

Cells were acquired on BD FACS Celesta and analyzed using FlowJo 9.6.

Activation Induced Marker T cell Assay: As previously described (4,5), PBMCs (1x10^6^ cells) were cultured in 96 well U-bottom plates containing 5% human AB serum (GemBio) in RPMI supplemented with penicillin/streptomycin and L-Glutamax. Prior to addition of megapools, cells were blocked with anti-CD40 mAb (Miltenyi Biotec) at 37$^{\circ}$C for 15 minutes. An equimolar amount of DMSO as a negative control, SARS-CoV-2 Spike megapool (1$\mu$g/mL), CMV megapool (1$\mu$g/mL), or staphylococcal enterotoxin B (SEB at 1$\mu$g/mL) was added to the cells. SARS-CoV-2 Spike megapools and CMV megapools were provided by the Sette lab (1,6). Cells were stimulated for 24 hours and then stained. Antigen-specific CD4^+^ T cells are defined as OX40^+^CD40L^+^CD4^+^ T cells. Antigen-specific CD8^+^ T cells are defined as CD69^+^41BB^+^CD8^+^ T cells.

Antibodies included: Live/Dead e780, CD19 (APC-eFluor 780, Clone HIB19), CD14 (APC-eFluor 780, Clone 61D3), CD16 (APC-eFluor 780, Clone CB16), CCR7 (AF700, Clone G043H7), 41BB/CD137 (APC, Clone 4B4-1), CXCR5 (BV421, Clone J25D4), CD45RA (BV570, Clone HI100), CD8a (BV650, Clone RPA-T8), CD4 (BV786, Clone OKT4), OX40 (FITC, Clone Ber-ACT35), CD40L/CD154 (PerCP-eFluor 710, Clone 24-21), CD69 (Pe-Cyanine7, Clone FN50).

Cells were acquired on BD FACS Celesta and analyzed using FlowJo 9.6.

1. Grifoni A, Weiskopf D, Ramirez SI, *et al.* Targets of T Cell Responses to SARS-CoV-2 Coronavirus in Humans with COVID-19 Disease and Unexposed Individuals. Cell 2020;181:1489-1501.e15.

2. Moderbacher CR, Ramirez SI, Dan JM, *et al.* Antigen-Specific Adaptive Immunity to SARS-CoV-2 in Acute COVID-19 and Associations with Age and Disease Severity. Cell 2020;183:996-1012.e19.

3. Dan JM, Mateus J, Kato Y, *et al.* Immunological memory to SARS-CoV-2 assessed for up to 8 months after infection. Science 2021;eabf4063.

4. Dan JM, Arlehamn CSL, Weiskopf D, *et al.* A Cytokine-Independent Approach To Identify Antigen-Specific Human Germinal Center T Follicular Helper Cells and Rare Antigen-Specific CD4+ T Cells in Blood. J Immunol Baltim Md 1950 2016;197:983–93.

5. Reiss S, Baxter AE, Cirelli KM, *et al.* Comparative analysis of activation induced marker (AIM) assays for sensitive identification of antigen-specific CD4 T cells. Plos One 2017;12:e0186998.

6. Grifoni A, Sidney J, Zhang Y, *et al.* A Sequence Homology and Bioinformatic Approach Can Predict Candidate Targets for Immune Responses to SARS-CoV-2. Cell Host Microbe 2020;27:671-680.e2.
