# Supplementary material for: Identification of Immunoglobulin G Autoantibody Against Malondialdehyde-Acetaldehyde Adducts as a Novel Serological Biomarker for Ulcerative Colitis
Source: Clin Transl Gastroenterol. 2022 Mar 14;13(4):e00469. doi: 10.14309/ctg.0000000000000469 (PMC9038499; doi:10.14309/ctg.0000000000000469)
Supplement: SUPPLEMENTARY MATERIAL [file ct9-13-e00469-s002.docx]

**Supplementary Figure Legends**

Supplementary Figure-1: Comparison of the blood levels of anti-MAA immunoglobulin isotypes in the IBD, non-IBD and controls: ELISA were done to determine the anti-MAA antibodies in the serum. A-C. Serum level of anti-MAA antibodies in IBD, non-IBD and controls.

Supplementary Figure-2: Comparison of the blood levels of anti-MAA immunoglobulin isotypes in the IBD: A-D. Serum/plasma level of anti-MAA IgM and IgA in adult and pediatric cohorts. Data were transformed into natural log scale.

Supplementary Figure-3: Logistic regression analysis for diagnostic performance of the anti-MAA immunoglobulins as biomarker for the detection of UC in adult cohort: A-B. ROC curve for UC vs HC, and C-D. ROC curve for CD vs HC.

Supplementary Figure-4: ROC curves analysis showing the diagnostic performance of anti-MAA immunoglobulins in pediatric cohort: A-B. ROC curve for UC vs HC, and C-D. ROC curve for CD vs HC.

Supplementary Table-1: Descriptive analysis for the IBD, IBS, and Celiac disease patients and controls.
