# Supplementary figures and images for: Identification of Immunoglobulin G Autoantibody Against Malondialdehyde-Acetaldehyde Adducts as a Novel Serological Biomarker for Ulcerative Colitis
Source: Clin Transl Gastroenterol. 2022 Mar 14;13(4):e00469. doi: 10.14309/ctg.0000000000000469 (PMC9038499; doi:10.14309/ctg.0000000000000469)

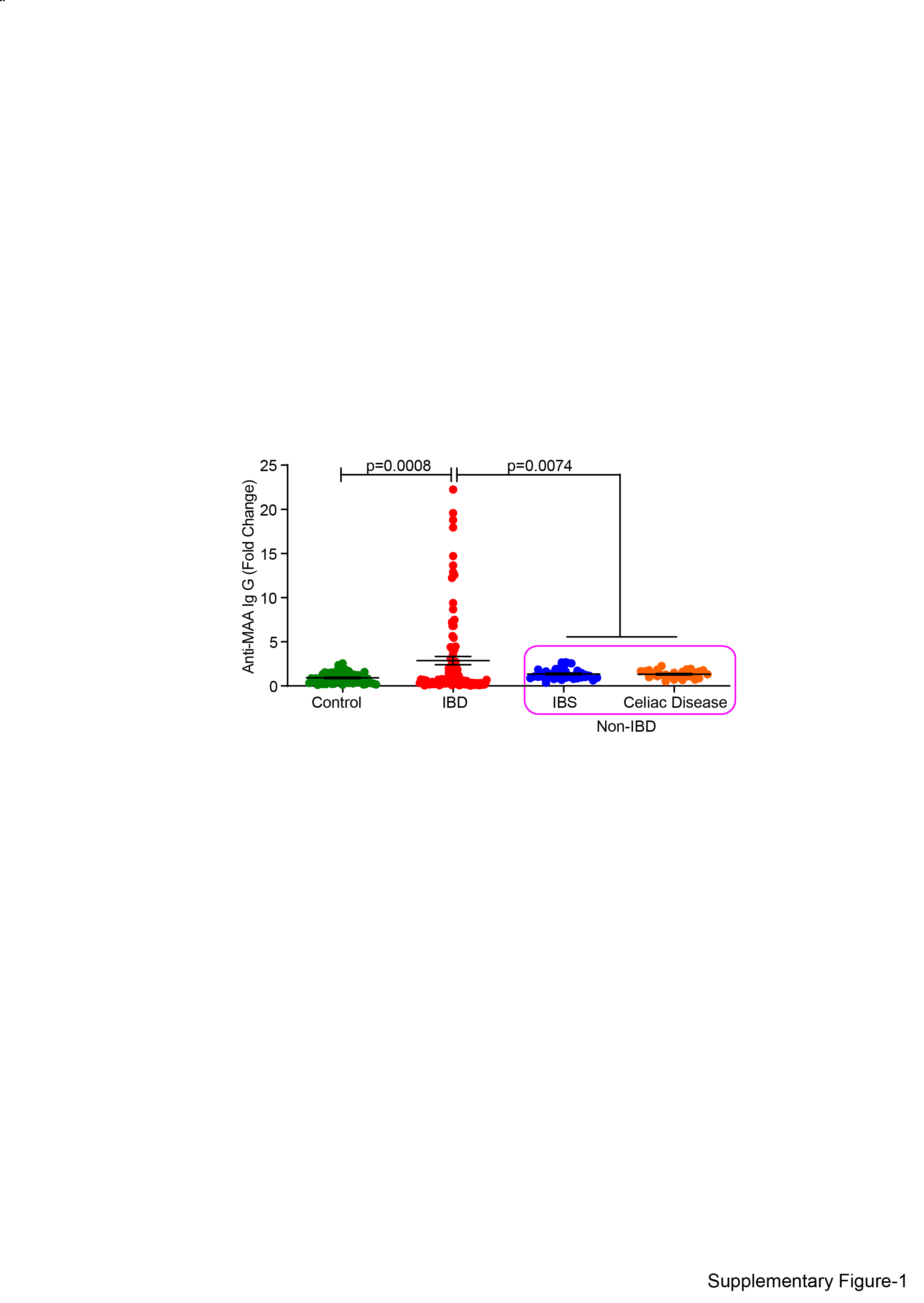

Supplement: SUPPLEMENTARY MATERIAL [file ct9-13-e00469-s004.jpg]

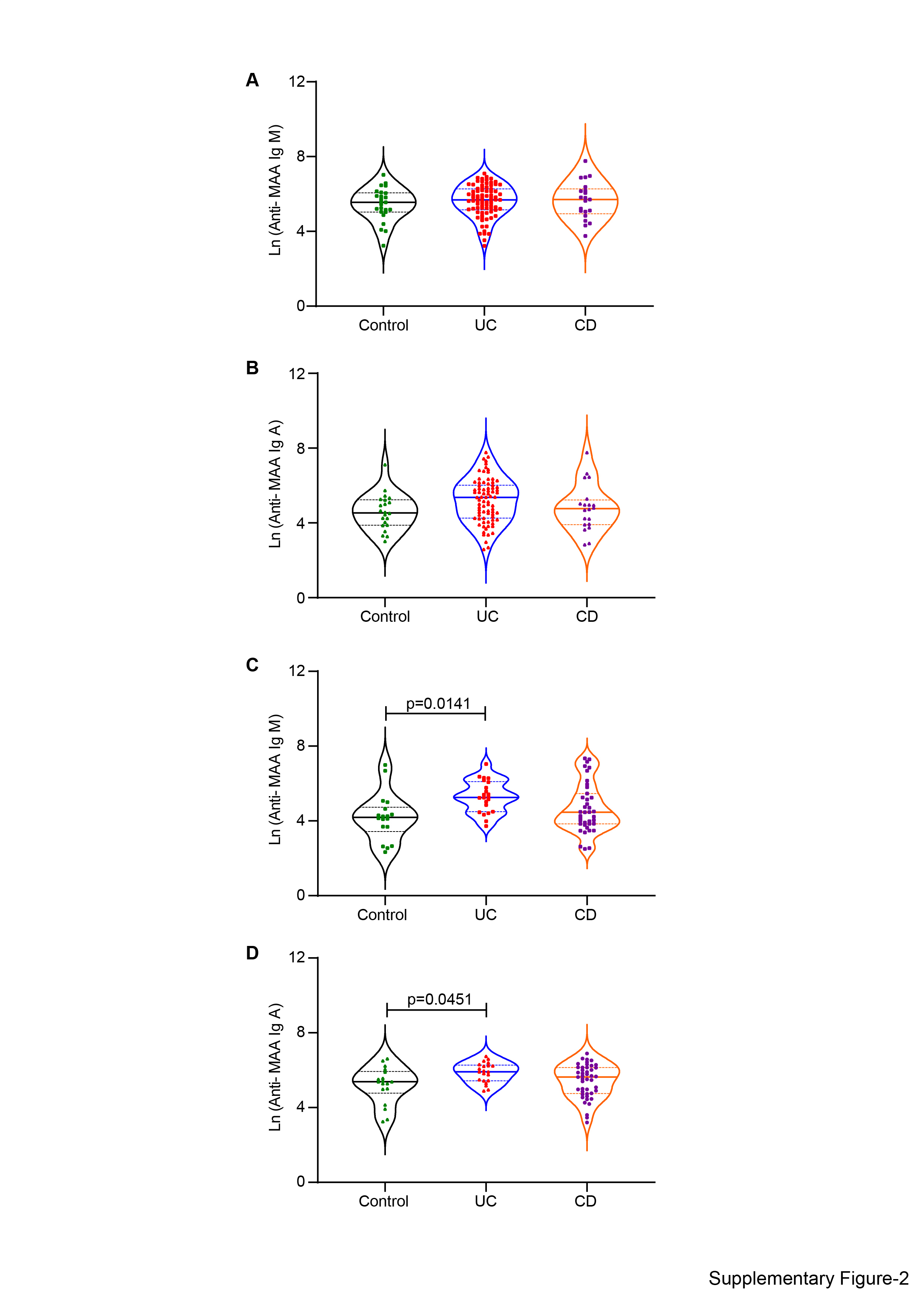

Supplement: SUPPLEMENTARY MATERIAL [file ct9-13-e00469-s005.jpg]

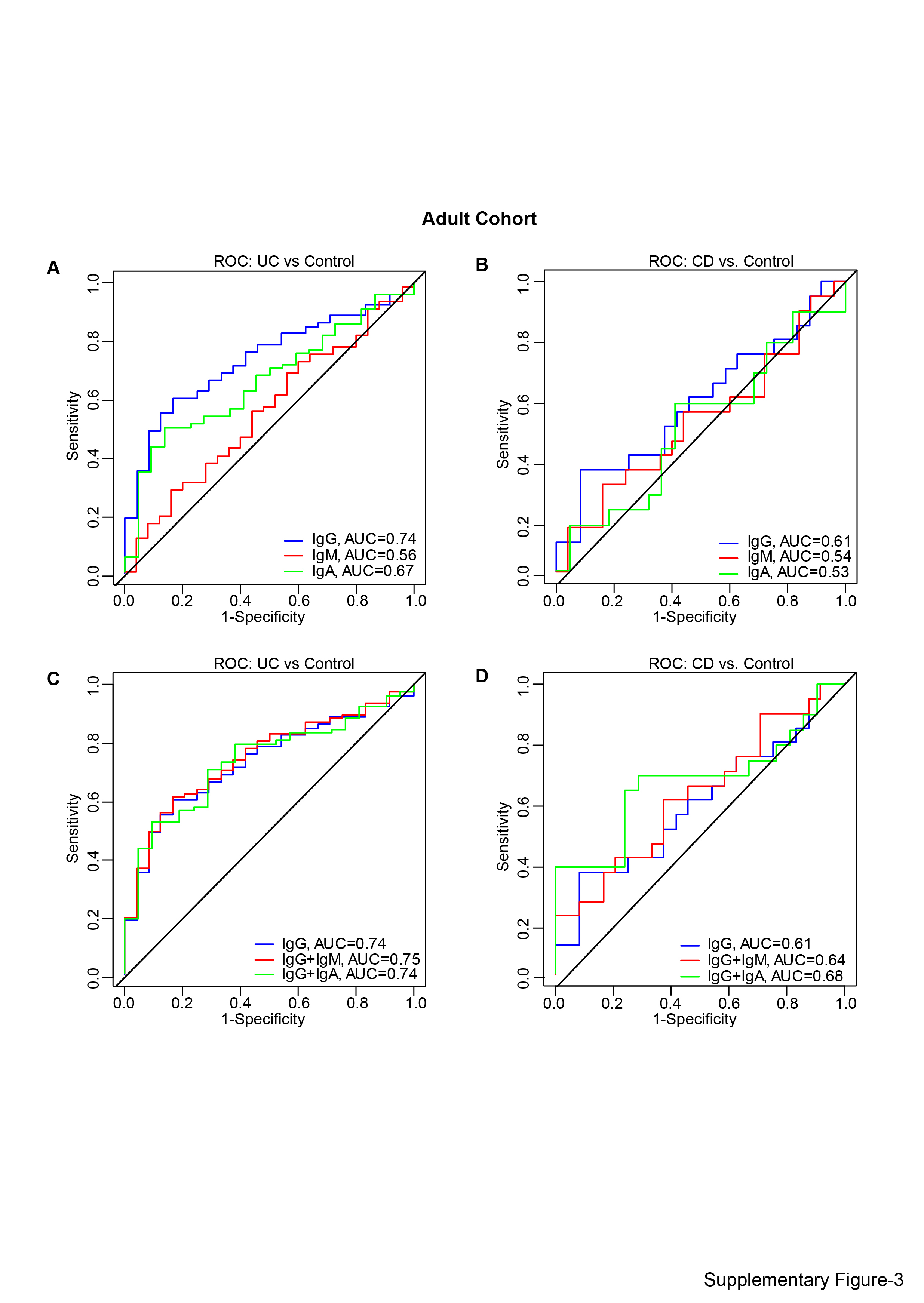

Supplement: SUPPLEMENTARY MATERIAL [file ct9-13-e00469-s006.jpg]

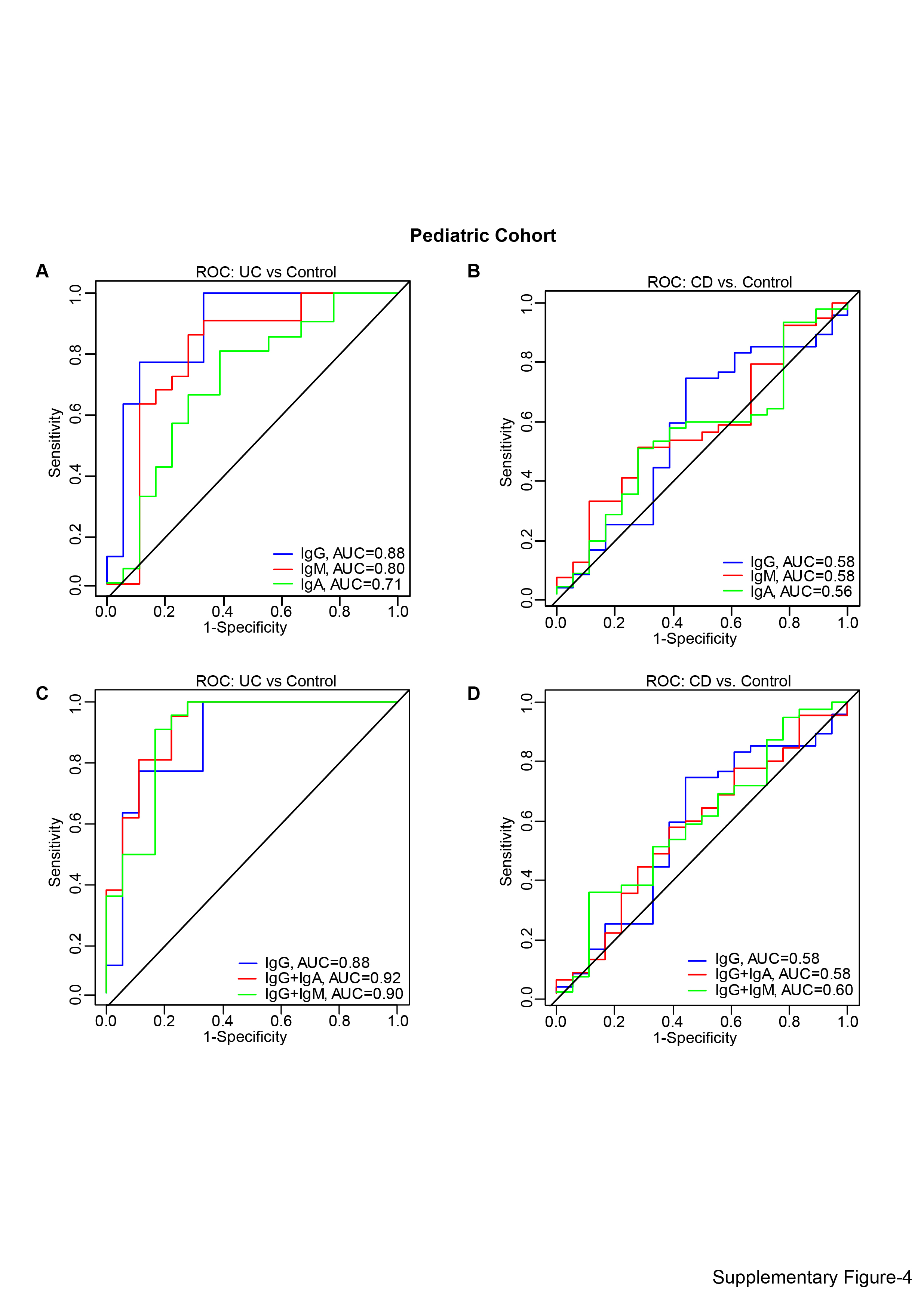

Supplement: SUPPLEMENTARY MATERIAL [file ct9-13-e00469-s007.jpg]
